# mGem: Fungal adhesins in *Candidozyma auris* confer unique fitness for skin colonization

**DOI:** 10.1128/mbio.03805-25

**Published:** 2026-03-09

**Authors:** Abishek Balakumar, Shankar Thangamani

**Affiliations:** 1 Department of Comparative Pathobiology, College of Veterinary Medicine, Purdue University 70731, West Lafayette, Indiana, USA; Georgia Institute of Technology, Atlanta, Georgia, USA

**Keywords:** *Candida auris*, adhesins, skin colonization, noscomial transmission

## Abstract

*Candidozyma auris* (formerly *Candida auris*) is an emerging multidrug-resistant fungal pathogen that causes life-threatening infections in humans. *C. auris* is distinct from other *Candida* species and exhibits exceptional capacity for skin colonization, resulting in nosocomial transmission and outbreaks of invasive infections. Fungal adhesins play a crucial role in skin colonization. With this perspective, we discuss the recent advances in the fungal adhesins of *C. auris* and how the divergence of adhesins in *C. auris* contributes to its unique fitness for skin colonization. We also discuss potential avenues to target fungal adhesins, which could pave the way for developing novel vaccine strategies and therapeutics to prevent skin colonization, nosocomial transmission, and invasive *C. auris* infections in humans.

## PERSPECTIVE

*Candidozyma auris* is an emerging multidrug-resistant fungal pathogen that persistently colonizes the skin of hospitalized patients and nursing home residents, resulting in nosocomial transmission and outbreaks of invasive fungal infections (1–3). It was recently classified as an urgent threat by the Centers for Disease Control and as a priority fungal pathogen by the World Health Organization (4, 5). Unlike other *Candida* species, which predominantly colonize the gastrointestinal tract, *C. auris* predominantly colonizes and persists in the skin for several months (6–10). *C. auris* not only colonizes the superficial skin surface but also enters the hair follicles and deep dermis (6, 11). Fungal load on human skin is a significant risk factor for subsequent infection and a source of transmission (1, 3). However, the factors that regulate *C. auris* skin colonization are not clear. Since fungal colonization facilitates *C. auris* nosocomial transmission and subsequent invasive infections, understanding the fungal adhesins that regulate *C. auris* skin colonization will open the door to developing novel therapeutics to prevent and treat this emerging fungal infection in humans. In this perspective, we discuss the recent discovery of fungal adhesins in *C. auris* and their role in skin colonization, as well as strategies to prevent it by targeting fungal adhesins.

## LINEAGE-SPECIFIC ADHESINS DELIVER UNIQUENESS TO *C. AURIS*

In *Candida* species, the cell wall proteins belonging to the ALS (agglutinin-like sequence) family, IFF/HYR (IPF family F/hyphally regulated) proteins, HWP family, and Epa family are the major families of adhesins involved in fungal adherence to host cells or inanimate surfaces (12, 13). However, the genetic progression in *Candida* species has expanded the adhesins with distinct domain architectures, featuring redundant or conserved adhesive functions across lineages. In *C. auris*, there are 12 adhesin proteins belonging to the ALS and IFF/HYR family, which have evolved independently through multiple chronological expansions as a result of functional diversification from other *Candida* species and do not exhibit precise homology within the genus (13). These adhesin proteins in *C. auris* have effector domains with heterogeneity in their adherence activity, resulting from a series of positive selections (14). In *C. auris*, the genes encoding ALS and IFF/HYR family adhesins are enriched in chromosomal regions that often undergo rearrangements and loss of subtelomeric areas (14, 15). This variability may confer *C. auris* strains with the capacity to differentially adhere to host niches, including skin.

**Fig 1:**
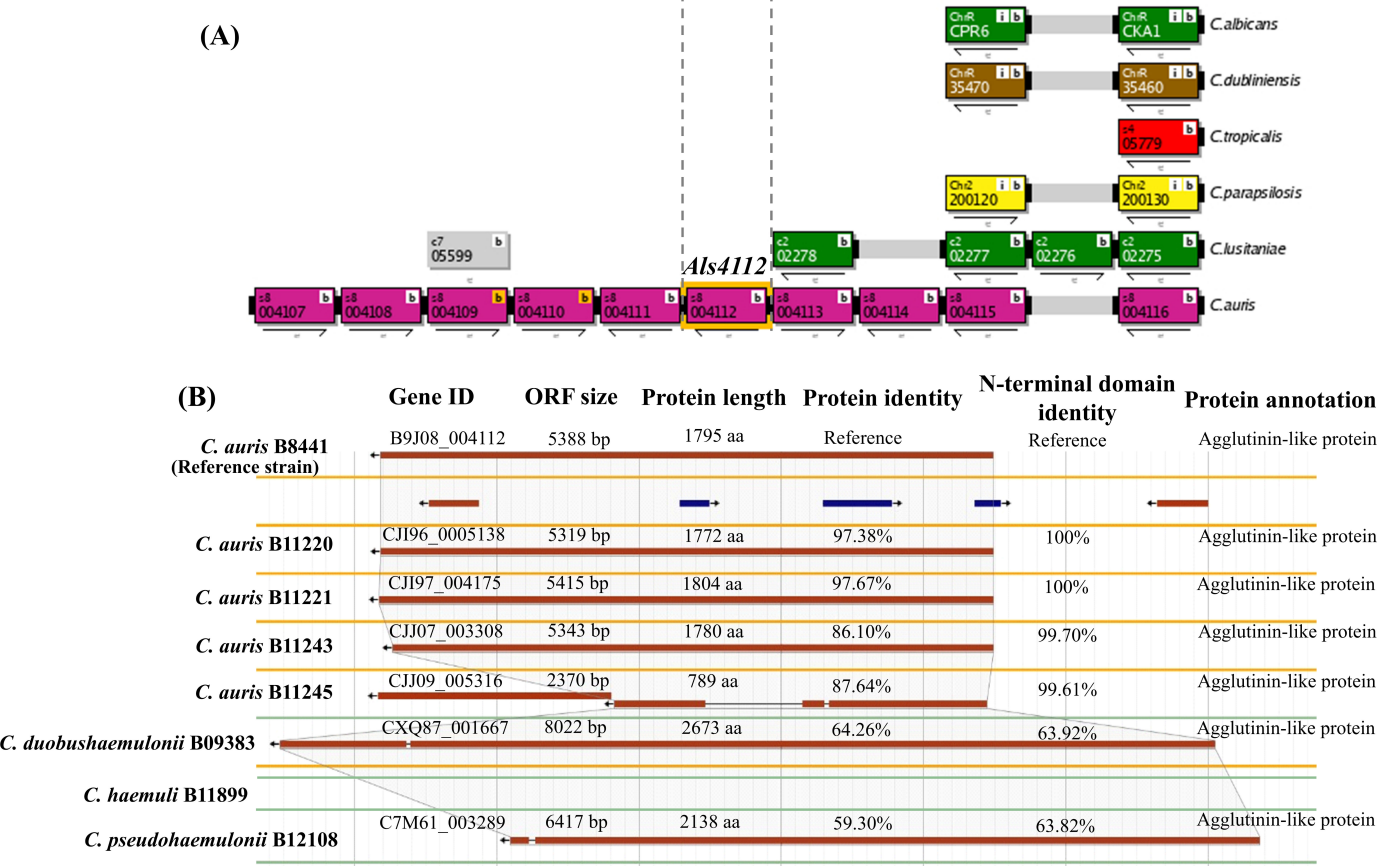
The molecular synteny of *ALS4112* in the *C. auris* B8441 strain in comparison to (**A**) genomic loci of the adjacent ORFs in distant *Candida* ancestors from the CTG clade and (**B**) the *ALS4112* orthologs within the *C. auris* clades and other closely related lineage-specific members.

The heterogeneity of adherence of fungal adhesins and their functional redundancy in colonization have been recently characterized in *C. auris*. The genetic screening with insertional mutants revealed that the fungal adhesins, such as Scf1 and Als4112, promote *C. auris* adhesion to skin (11, 13). The *SCF1* and *ALS4112* in *C. auris* are lineage-specific, and their orthologs are shared only among a few members of the *Candidozyma haemuli* species complex (formerly *Candida haemulonii*) (13, 16). The orthologs of *SCF1* were detailed previously (13). Here, we show the orientation of adjacent ORFs to *ALS4112* in distant *Candida* ancestors and the *ALS4112* orthologs shared by *Candidozyma* species (Fig. 1). Both *SCF1* and *ALS4112* are nonexistent in other *Candida* species, which defines the phylogenetic distinction of the adhesins in *Candidozyma* (both *C. auris* and *C. haemuli* complex) from other CTG clades (16–18) (Fig. 1A).

Although *C. haemuli* shares a functional homolog of Scf1 with potential adherence activity similar to that of *C. auris*, its expression is not regulated in clinical isolates of *C. haemuli* (13). On the other hand, the orthologs of *ALS4112* shared by all the geographically stratified clades of *C. auris* are expanded only from *C. pseudohaemuli* and *C. duobushaemuli* (Fig. 1B). All these ORFs encode an ALS adhesin. However, the protein and N-terminal domain sequence identity of Als4112 reveals that the protein homology is highly conserved among the *C. auris* clades but evolved from the *C. pseudohaemuli* and *C. duobushaemuli* (Fig. 1B). This independent expansion and divergence of Als4112 in *C. auris,* with distinction in N-terminal amino acid sequences, may contribute to skin colonization and distinguish them from the members of the *C. haemuli* complex, which are poor skin colonizers and do not cause major outbreaks in the hospital like *C. auris* (19). However, the gene expression of *ALS4112* orthologs in *C. pseudohaemuli* and *C. duobushaemuli* is still unknown.

## SCF1 AND IFF4109 PROMOTE BIOFILM FORMATION IN *C. AURIS* FOR SKIN COLONIZATION

**Fig 2:**
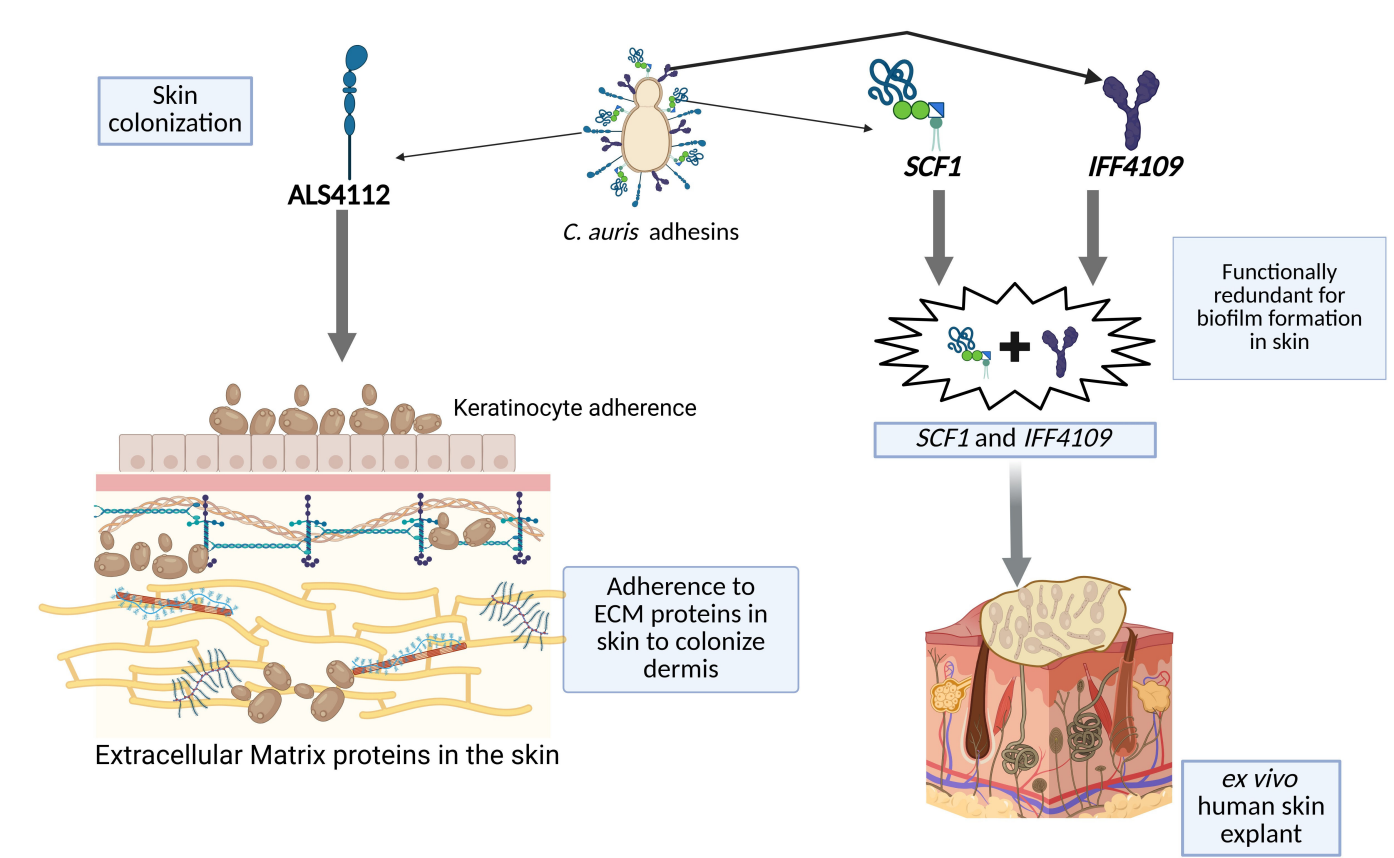
*C. auris* adhesins regulate fungal colonization on skin.

The genetic screening of *C. auris* enabled the discovery of Scf1, an adhesin with a unique adhesion mechanism that binds to polymer surfaces (13). The identified adhesin Scf1 plays a role in surface colonization in *C. auris* as described previously (13). However, the Scf1 in *C. auris* is also required for biofilm formation and skin colonization, which is the focus of this review. The Scf1 promotes biofilm formation in *C. auris in vitro* and *in vivo* (13), and the gene expression is highly upregulated in both conditions (20). The biofilm formation in *C. auris* is strain-specific, and the AR0382 strain forms a robust biofilm, while the AR0387 strain forms a poor biofilm. The expression of *SCF1* in these strains correlates with their biofilm-forming capacity and skin colonization potential (13, 20). *SCF1* plays a complementary role and is functionally redundant with *Candida* adhesin *IFF4109* for biofilm formation. Both adhesins contribute to adhesion through distinct mechanisms and are not regulated by one another. However, the mechanism of biological surface association by Scf1 was due to the biofilm-forming capacity together with Iff4109 (13) (Fig. 2). Deletion of both *SCF1* and *IFF4109* in *C. auris* only ablates the biofilm formation, and deletion of *SCF1* or *IFF4109* alone does not affect the biofilm. However, the overexpression of *SCF1* alone restored the biofilm formation capacity in the poor biofilm-forming strain AR0387, similar to that of the high biofilm-forming strain AR0382 (13). A similar pattern was observed in the aspect of skin colonization. The *scf1*∆ *Iff4109*∆ mutants in AR0382 had significantly decreased colonization in *ex vivo* human skin explants and *in vivo* murine skin. Furthermore, the overexpression of *SCF1* in the AR0387 restored the skin colonization potential in *ex vivo* human skin explants and *in vivo* murine skin, similar to the AR0382 strain (13). This indicates that Scf1, along with Iff4109, in *C. auris* promotes biofilm formation and is necessary for skin colonization (Fig. 2). *C. auris* forms a high-burden biofilm in the skin microenvironment, which mimics the physiological condition of the skin (21). The role of Scf1 and Iff4109 in skin colonization may be indirectly due to biofilm formation.

## ALS4112 PROMOTES ROBUST SKIN COLONIZATION

*C. auris* colonizes both superficial and deep skin surfaces and resides in and around the hair follicles of the skin, which are composed mainly of keratinocyte epithelium (6). The recent study identified that the fungal adhesin Als4112 mediates *C. auris* adhesion to skin keratinocytes (11) (Fig. 2). Als4112 is highly conserved among the major clades of *C. auris* at the N-terminal and C-terminal regions and in the number of tandem repeats (11). The gene expression of the *ALS4112* gene is dynamic among all five geographical clades, and it correlates with the keratinocyte adherence activity (11).

Als4112, belonging to the ALS family of fungal adhesins, is a glycosylphosphatidylinositol (GPI)-anchored protein in the cell wall. The ALS adhesins exhibit adhesive activity through the N-terminal domain, which comprises a peptide-binding cavity (PBC) that mediates adhesion to host cells (22). The PBC of the ALS adhesins has a tendency to recognize and bind to diverse peptide sequences in the host cells and matrix proteins in the tissues. The members of ALS adhesins can recognize the specific C-terminal carboxylic acid of the incoming host peptides and mediate adhesion through electrostatic interactions with the invariant lysine residues in the N-terminus of the ALS protein (23). The hypervariability of amino acid sequences present in the N-terminal domain of ALS adhesins results in functional diversity, enabling them to bind to diverse host substrates (24). Furthermore, the adhesins in *C. auris* after multiple independent expansions from *Candida* species led to functional diversification of the N-terminal effector domain, followed by a series of positive selections resulting in variation of adhesion to the different niches, including skin (14).

The skin tissue is composed of complex extracellular matrix proteins (ECM) for structural integrity (25). Mechanistically, the authors examined whether Als4112 binds to ECM proteins that promote skin colonization (11). Screening of 35 distinct ECM proteins that are abundant in skin tissue revealed that *C. auris* has high efficiency binding to laminin in the skin (11) (Fig. 2). The competitive binding of *C. auris* to the laminin in the skin was significantly reduced in the *Als4112*Δ strain, demonstrating that Als4112 is critical for ECM binding (11). In the murine skin, the epicutaneous colonization of the *Als4112*Δ strain after 2 days had a significantly lower skin fungal burden than the WT strain, and the reintegration of *ALS4112* in the *Als4112*Δ strain reverted the fungal burden to a level similar to that of the WT strain. A similar trend was observed in the *ex vivo* human skin model. These findings suggest that the adhesin Als4112 in *C. auris* is required for efficient skin colonization, and this fungal factor confers skin tropism to this emerging fungal pathogen (11). In *Candida* species, the genomic loci of ALS family genes exhibit a high level of variability in transcriptional regulation and gene copy numbers (26). The previous study indicates that the clinical isolates of *C. auris* are subjected to genomic amplification of *ALS4112* due to copy number variation (CNV) that occurs in the unstable subtelomeric region (27). The Als4112 also promotes cell aggregation, which enhances biofilm formation and, in turn, increases the ability of *C. auris* to colonize the murine skin *in vivo* (27). The evidence suggests that Als4114 directly interacts with the Scf1 through homophilic interactions and complementary binding of these adhesins between cells leading to cell-cell aggregation (20). Collectively, Als4112 can directly bind to keratinocytes and ECM proteins in skin tissue and also enhances fungal cell aggregation, thereby promoting *C. auris* skin colonization. However, a recent study identified that cell-cell aggregation could be Als4112-dependent or independent in *C. auris* (28). Future studies are needed to understand the other mechanisms by which Als4112 regulates skin colonization.

## TARGETING FUNGAL ADHESINS COULD BE A NOVEL STRATEGY AGAINST *C. AURIS*

The adhesins are crucial factors in determining fungal colonization of different host niches. Targeting specific fungal adhesins may be a promising future direction for developing novel vaccine or drug strategies to prevent and treat *C. auris* infection. Among the fungal vaccinations, NDV-3A is the most extensively studied and is currently in clinical trials (29). The NDV-3A immunization in mice prevents the ability of *C. albicans* to adhere and invade endothelial cells and the colonization of jugular vein catheters in mice (30). The NDV-3A vaccine, which encompasses the N-terminus of the *C. albicans* Als3 protein, was previously studied to target the ALS member adhesins in *C. auris* (29). *C. auris* ALS proteins have homologs of approximately 30% identity and 50% similarity to the *C. albicans* Als3 protein, and anti-Als3p antibodies can recognize and adhere to all four clades, suggesting universal Als3p homolog distribution. The anti-Als3p sera inhibited the ability of *C. auris* to form biofilms and enhanced opsonization-mediated killing by macrophages (29). Similarly, the anti-mouse sera of the NDV-3A vaccine immunized mice significantly inhibited the keratinocyte adherence of *C. auris* (11). However, in *C. auris*, Als4112 and Scf1 are specific adhesins mediating skin colonization through keratinocyte adherence and biofilm formation, and their protein homology and N-terminal domain sequences are highly conserved among all the clades (11, 13). The development of vaccination strategies targeting the N-terminus of these adhesins could be a promising future approach to specifically target *C. auris* to prevent colonization. In addition to vaccines, compounds/drugs that target fungal adhesins could be developed to prevent *C. auris* skin colonization. In *Aspergillus fumigatus*, the development of synthetic multivalent fucosides targeting the FleA lectin inhibited the adherence of fungal conidia to pneumocytes (31, 32). However, currently, there are no molecules available to target the *Candida* adhesins. Future development of anti-adhesive compounds targeting Als4112 and Scf1 could lead to a new therapeutic venue to combat *C. auris* colonization.

*C. auris* has differential binding specificity to ECM proteins in the skin. It binds robustly to laminin and poorly to collagen I or III. A study shows that coating collagen I or III on plastic surfaces mitigates the adherence and biofilm formation of *C. auris* (11). This reduction in adherence was observed in all four clades, indicating a broad spectrum of adherence inhibition in *C. auris*, despite high clade-level variability. A similar pattern was observed when the polyethylene catheters were coated with collagen I or III (11). The collagen III-coated central venous catheters, when inserted into the jugular veins of rats, significantly reduced *C. auris* colonization on the luminal catheter surface after 24 h of infection (11). These findings highlight the potential of specific collagen coatings as a novel approach to prevent *C. auris* adherence and biofilm formation on medical devices. Collectively, targeting the fungal adhesins will open a new direction for novel vaccine strategies and antifungal therapeutics to prevent and treat *C. auris* colonization in the skin, thereby preventing nosocomial transmission and outbreaks of systemic infections in immunocompromised patients.
